# Cryo-electron microscopy in the study of virus entry and infection

**DOI:** 10.3389/fmolb.2024.1429180

**Published:** 2024-07-24

**Authors:** Moumita Dutta, Priyamvada Acharya

**Affiliations:** ^1^ Duke Human Vaccine Institute, Durham, NC, United States; ^2^ Department of Surgery, Durham, NC, United States; ^3^ Department of Biochemistry, Duke University, Durham, NC, United States

**Keywords:** cryo-EM, cryo-ET, HIV-1, SARS-CoV-2, bacteriophage, 3D reconstructions, structure-based design, vaccine development

## Abstract

Viruses have been responsible for many epidemics and pandemics that have impacted human life globally. The COVID-19 pandemic highlighted both our vulnerability to viral outbreaks, as well as the mobilization of the scientific community to come together to combat the unprecedented threat to humanity. Cryo-electron microscopy (cryo-EM) played a central role in our understanding of SARS-CoV-2 during the pandemic and continues to inform about this evolving pathogen. Cryo-EM with its two popular imaging modalities, single particle analysis (SPA) and cryo-electron tomography (cryo-ET), has contributed immensely to understanding the structure of viruses and interactions that define their life cycles and pathogenicity. Here, we review how cryo-EM has informed our understanding of three distinct viruses, of which two - HIV-1 and SARS-CoV-2 infect humans, and the third, bacteriophages, infect bacteria. For HIV-1 and SARS-CoV-2 our focus is on the surface glycoproteins that are responsible for mediating host receptor binding, and host and cell membrane fusion, while for bacteriophages, we review their structure, capsid maturation, attachment to the bacterial cell surface and infection initiation mechanism.

## 1 Introduction: from blobology to high resolution structural biology

Electron microscopy has a long history of being used as a diagnostic tool to identify new infectious agents, study their structures, and understanding virus-cell interaction (Friedlaender et al., 1955; Roingeard, 2008; Goldsmith and Miller, 2009). Since its invention, the electron microscope has not only been used for viral diagnoses but also in biomedical research to examine the structure of tissues, cells, organelles, and macromolecular complexes, and in pathogenesis studies. Biological samples, during their visualization under an electron microscope, suffered damage due to dehydration and staining. Therefore, rapid freezing of unstained biological samples was developed during the 70s–80s (Dubochet et al., 1988). With the development of sophisticated cryo-electron microscopy (cryo-EM) technique, detailed structures of viruses were determined in their native state. In its early days, cryo-EM structures mostly looked like blobs due to low-resolution imaging, hence the moniker “blobology”. However, the immense potential of cryo-EM was recognized, and with investment of time and resources, cryo-EM has come a long way with steadily improving microscopes, and improved methods for sample purification, specimen preparation, data collection and image analysis (Milne et al., 2013; Thompson et al., 2016; Saibil 2017; Dandey et al., 2018; Noble et al., 2018). Cryo-EM has now emerged as a primary structural biology technique and was recognized as the method of the year in 2015 (2016). The development of direct electron detectors transformed the cryo-EM field, and the world witnessed the resolution revolution that culminated in the Nobel Prize in Chemistry in 2017 being awarded to Jacques Dubochet, Joachim Frank and Richard Henderson for developing freezing method of biomolecules, image-processing software and first 3D model and atomic structure of a protein, respectively (Kühlbrandt, 2014a; Cressey and Callaway, 2017). Thereafter, the number of entries in the Electron Microscopy Data Bank (EMDB), the public archive for three-dimensional EM maps, has rapidly increased (Patwardhan, 2017).

Advances in cryo-EM have improved the quality of imaging and analysis. Moreover, the classification schemes used in cryo-EM image processing have made it possible to separate multiple conformations and compositions of macromolecular complexes within the same dataset. This sets cryo-EM apart from many other structural techniques that rely on homogeneous sample preparations, making it versatile and useful for studying complex samples (Fernández et al., 2013; Kühlbrandt, 2014b; Scheres, 2016). Computational techniques for enhancing local resolution can address issues of lowered resolution due to local sample heterogeneity and conformational flexibility (Kucukelbir et al., 2014; Punjani et al., 2017; Grant et al., 2018). Noticeably, more countries are getting involved in this cutting-edge technique to tackle future threats of infectious diseases. Cryo-EM has been widely used for understanding Zika, Nipah, Ebola, Influenza, SARS, MERS, Langya, Lassa, Dengue, Chikungunya, HIV, and other viruses (Harris et al., 2006; Lyumkis et al., 2013, Sirohi et al., 2016; Yuan et al., 2017; Song et al., 2018, Su et al., 2018; Basore et al., 2019; Dang et al., 2019; Stass et al., 2019, Katz et al., 2022; Guo et al., 2024). With advances in cryo-EM methodology, it is now possible to obtain near-atomic resolutions of viruses and their spike structures, their interaction with host cell receptors, virus entry, assembly and antibody-mediated neutralization (Casasnovas, 2013; Stass et al., 2019; Burton, 2023). All these discoveries can collectively contribute to the development of new vaccines and antiviral therapies.

In this review, we highlight the contribution of two cryo-EM imaging modalities, SPA and cryo-ET, in understanding the protein structures and life cycles of three viruses of relevance to human health, of which two–HIV-1 (Mak and de Marco, 2018; Graham and Zhang, 2023) and SARS-CoV-2 (Zhang et al., 2021a) cause disease in humans, whereas the third, bacteriophages, infects bacteria and has been harnessed for biotechnological applications, and for combatting drug-resistant bacteria (Jo et al., 2023).

## 2 Contribution of Cryo-EM and its advances in studying virus-mediated infectious diseases

**FIGURE 1:**
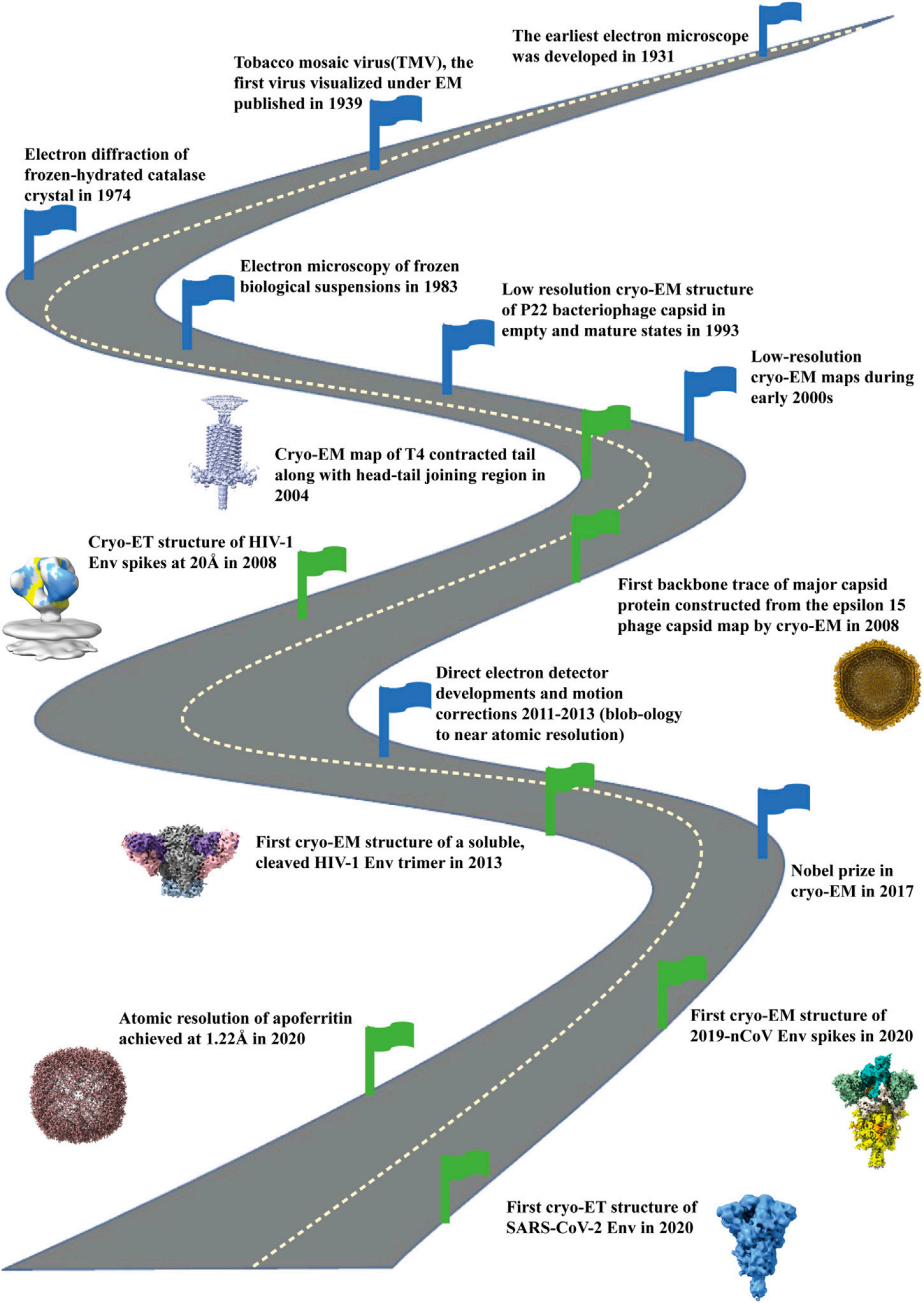
Road map of development of cryo-electron microscopy field. Milestones indicated with blue flags starting from development of electron microscope (1931), visualization of first virus under EM (1939), electron diffraction of frozen, hydrated catalase crystals (1974) and development of electron microscopy of frozen biological samples (1983). Then low resolution cryo-EM maps were reconstructed during early 2000. With the development of direct electron detectors around 2011-2013 there was an upsurge of high resolution cryo-EM structures. In 2017 the Nobel Prize in Chemistry was given to the three pioneers (Jacques Dubochet, Joachim Frank, Richard Henderson) for the development of structure determination by cryo-electron microscopy. By the year 2020 atomic resolution (1.22Å) structure was achieved for apoferritin (EMD-11638) by cryo-EM. Green flags indicate the important milestones of the contributions of cryo-EM in bacteriophages (EMD-1086; 5003), HIV Envelope (EMD-5779; 5019) and SARS-CoV2 spike (EMD-21375; 11494) structure determination, the three topics that are the focus of this paper.

Electron microscopy has been at the forefront for fast detection of any infectious agent and its morphology (Hazelton and Gelderblom, 2003). With the development of cryo-EM, the structures of viruses, their interaction with the host, and disease progression have been successfully studied, with technological advances in the cryo-EM field enabling near-atomic structural insights having transformative impact on molecular and cellular structural biology. (Jiang and Tang, 2017; Creekmore et al., 2021; Graham and Zhang, 2023). Pathogenic viruses are infectious agents that exist in various size and shape in the virosphere. Pathogenic enveloped viruses have in common a membrane-embedded spike glycoprotein responsible for host-receptor binding to begin the infection process. Therefore, the spike protein is an important target to study in detail by cryo-EM (Dokainish et al., 2022). The resolution revolution resulted in an atomic-level understanding of many surface proteins of enveloped pathogenic viruses, thus enabling structure-based drug discovery and vaccine design (Graham et al., 2019; Kim and Jung, 2021). Figure 1 highlights the timeline of developments in cryo-EM technology juxtaposed with breakthroughs in our understanding of the bacteriophage capsid structure, capsid maturation, tail contraction, and surface glycoproteins of HIV-1 and SARS-CoV-2 (Leiman et al., 2004; Jiang et al., 2008; Liu et al., 2008; Lyumkis et al., 2013; Ke et al., 2020; Nakane et al., 2020; Wrapp et al., 2020). Of the two modalities of cryo-EM that have commonly been used to study the structures of viral proteins, single particle analysis (SPA) is the most popular cryo-EM technique for determining atomic resolution structures of “spike” proteins of enveloped viruses (Zhou, 2014; Ward and Wilson, 2017), while cryo-electron tomography (cryo-ET) provides *in situ* information within a native cellular context, including the distribution of proteins, the presence of any transient conformational changes, viral assembly, and maturation mechanism within the host (Subramaniam et al., 2007; Liu et al., 2010; Ke et al., 2020).

## 3 Structural studies on the HIV-1 Env

### 3.1 Contribution of cryo-EM/cryo-ET in advancing the knowledge of HIV-1 structural biology

#### 3.1.1 HIV structure

Human immunodeficiency virus-1 (HIV-1) is the causative agent of acquired immunodeficiency syndrome (AIDS). HIV-1 has a lipid envelope consisting of a bilayer membrane and trimeric envelope (Env) glycoprotein are embedded onto the membrane (Blumenthal et al., 2012). HIV-1 Env is composed of a receptor binding gp120 subunit and a fusion mediating gp41 subunit that contains the fusion peptide. Env plays a pivotal role in virus attachment to the host cell surface and in initiating the infection process through the fusion of the viral and host cell membrane (Xiao et al., 2021a). Several Env defense mechanisms, including a rapidly mutating genome that introduces antigenic variation, conformational masking and shielding of antigenic sites by long variable loops, have challenged the development of an effective HIV-1 vaccine (Haynes et al., 2023). However, critical regions that bind to the primary HIV-1 receptor CD4, and co-receptors, remain conserved and are among the primary targets of vaccine and drug development efforts.

Structural determination of Env began with a crystal structure of the gp120 core in complex with the primary HIV-1 receptor CD4 and the fragment antigen binding (Fab) of a coreceptor mimetic antibody 17b that was solved in the ‘90s (Kwong et al., 1998), followed by several other structures of gp120, unliganded or bound to antibodies and receptors. While these structures provided detailed views of gp120 domain organization, its internal architecture and epitopes targeted by antibodies, they were limited their utility in informing Env biology that required the context of the trimer. X-ray crystal structures of soluble HIV-1 Env ectodomains were later determined (Julien et al., 2013; Pancera et al., 2014; Kwon et al., 2015; Stewart-Jones et al., 2016) and while these structures provided information in the Env trimer context, obtaining well diffracting crystals of new complexes remained a bottleneck that hindered throughput.

#### 3.1.2 Cryo-ET of HIV-1 native spikes

**FIGURE 2:**
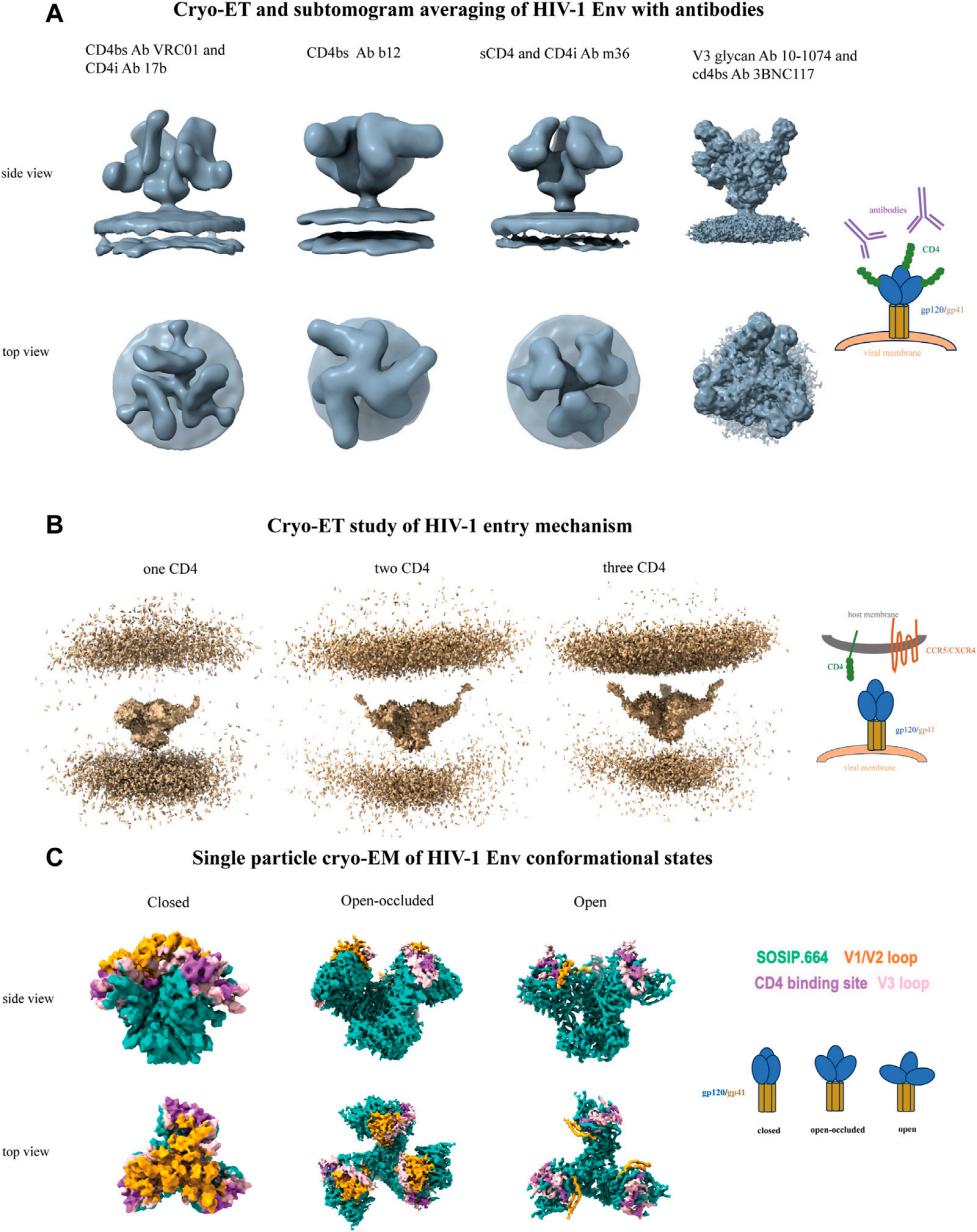
Cryo-ET and single particle cryo-EM of HIV-1 Env. **(A)** From left to right, CD4-binding site antibody VRC01 and CD4-induced antibody 17b (EMD-5461), CD4-binding site antibody b12 (EMD-5018), sCD4 and CD4-induced antibody m36 (EMD-5554), and V3-glycan antibody 10-1074 and CD4-binding site antibody 3BNC117 (EMD-21413). **(B)** Cryo-ET study of HIV-1 entry mechanism. From left to right, One (EMD-29292), two (EMD-29293) and three (EMD-29294) CD4 molecules sequentially bound to native HIV-1 Env on membrane brings the host and viral membranes close to each other to initiate the fusion process. **(C)** Single particle cryo-EM of HIV-1 Env conformational states. From left to right, closed (EMD-8714), open-occluded (EMD-25878, bound antibody not shown), open (EMD-8713, bound CD4 and antibody not shown) conformational states are shown with the Env colored sea green, V1V2 loop colored orange, V3 loop colored pink and CD4 binding site colored medium orchid. Each panel includes a schematic representation.

Electron microscopy provides a methodological platform for determining structures of biomolecules without the bottleneck of obtaining well diffracting crystals. Cryo-ET structures of SIV/HIV-Env spikes on intact virus membrane determined in early 2000s provided the first views of the HIV-1 Env on the virion surface (Zanetti et al., 2006; Zhu et al., 2006). These were subsequently followed by several cryo-ET structures of Env, either unliganded or in complex with receptors and antibodies, with better resolution enabled by technical advances in microscopic and sample preparation techniques (Figure 2A). These structures shed light on the mechanism of closed-to-open conformational change in the native, membrane-associated Env, (Liu et al., 2008; Tran et al., 2012; Meyerson et al., 2013; Li et al., 2020). Cryo-ET analysis on Env performed in both mature and immature HIV-1 particles provided information on the location and arrangement of structural proteins, e.g., radial arrangement of Gag structural protein along with hexameric capsid (CA) protein stabilization by bundle of six spacer element 1(SP 1) helices, and formation of fullerene cone shaped mature HIV-1 capsid in which capsid-protein hexamers and pentamers form a closed hexagonal surface lattice, tubular assembly of HIV-1 Gag derived protein reveals structural arrangement of C-terminal domain of CA and region which is essential for virus assembly located downstream of CA, tertiary and quaternary structural arrangement for viral assembly (Wright et al., 2007; Zhao et al., 2013; Bharat et al., 2014; Schur et al., 2015).

The HIV-1 assembly and its release in intact human cells suggested that the organization of the Gag protein occurs in the assembly site in immature HIV-1 with no further changes in arrangement. Proteolytic maturation results in two different hexameric lattice rearrangements of the HIV-1 matrix (MA) between immature and mature virions. The membrane-bound MA lattice plays a significant role in mature virions. Env on immature HIV-1 virion structure gives an idea about the Env positioning relative to Gag hexameric arrangement. The structural analysis of Env on mature HIV-1 viral particles revealed substantial glycan shielding, flexibility in the gp41 stalk that exposes the crucial epitopes present in FP, gp120/41 interface, and MPER regions differently and leads to structural variations. (Carlson et al., 2010; Qu et al., 2021; Mangala Prasad et al., 2022). Subsequent cryo-ET studies have increased our knowledge of HIV-1 entry and fusion. These include direct visualization of the hypothesized pre-hairpin intermediate of HIV-1 Env (Ladinsky et al., 2020), visualization of the contact between T-cells and SIV/HIV-1 during viral entry (Sougrat et al., 2007), and studies on HIV-1 and CD4, both on the membrane, showing three CD4 molecules gradually bind to Env through cluster and ring formation ultimately bringing both membranes closer and the transition of Env to an open state (Li et al., 2023) (Figure 2B).

#### 3.1.3 High-resolution cryo-EM structural studies of HIV-1 SOSIP

The development of stabilized HIV-1 Env ectodomain trimers (Sanders et al., 2002; Sanders et al., 2013a; Sliepen et al., 2019) synergized with the cryo-EM resolution revolution to turbo charge HIV-1 Env structural biology, making it faster and easier to obtain high resolution structures of the HIV-1 Env trimer (Lyumkis et al., 2013; Lee et al., 2016; Ozorowski et al., 2017). Overall features of Env trimer embedded in virions surface showed remarkable similarities with the structures of the engineered soluble trimers (Harris et al., 2011; Torrents de la Peña et al., 2019; Li et al., 2020). Cryo-EM has enabled high resolution visualization of Env conformational states that are relevant for viral entry and for antibody access to sites of vulnerability. The HIV-1 Env has a prefusion, closed state in which the gp41 helices that are anchored to the viral membrane act as a support to rearrange other parts of the spike when it changes to the receptor-bound open conformation (Figure 2C). HIV Env binding to CD4 induces extensive conformational changes in Env that eventually opens the Env and exposes the co-receptor binding sites (Ozorowski et al., 2017). Other partially open intermediates have been observed (Figure 2C) that open new avenues for the development of therapeutics to prevent viral infection (Scharf et al., 2015; Yang et al., 2022; Dam et al., 2023).

**FIGURE 3:**
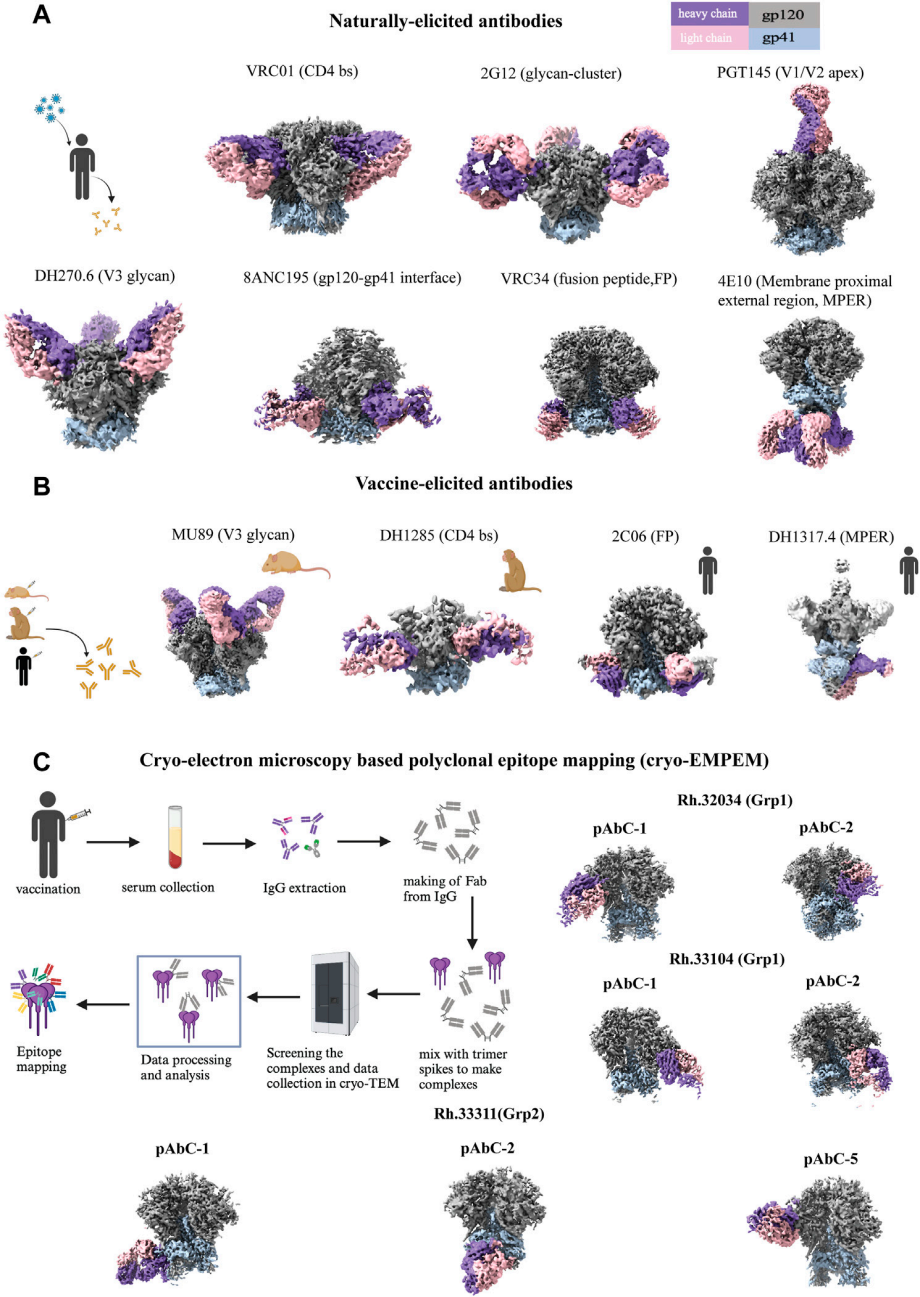
High resolution cryo-EM structures of HIV SOSIP Env with antibodies. **(A)** Naturally-elicited antibodies in complex with HIV SOSIP Env spike: From left to right (first row), CD4 binding site Ab VRC01 (EMD-40281), glycan cluster Ab 2G12 (EMD-20224), V1/V2-apex directed Ab PGT145 (EMD-29288). From left to right (second row), V3-glycan targeted Ab DH270.6 (EMD-20818), gp120-gp41 interface Ab 8ANC195 (EMD-0485), fusion peptide-targeting Ab VRC34 (EMD-28617), membrane proximal external region targeted Ab 4E10 (EMD-25045). **(B)** Vaccine-elicited antibodies in complex with HIV SOSIP Env spike: From left to right (first row), V3-glycan mouse Ab MU89 (EMD-27706), CD4 binding site macaque Ab DH1285 (EMD-41823), FP-targeting human Ab 2C06 (EMD-29725), MPER targeted human Ab DH1317.4 (EMD-44246). **(C)** Cryo-EM based polyclonal epitope mapping (cryo-EMPEM): From left to right (first row), polyclonal antibodies were selected from rhesus macaques Rh.32034 (Grp1): pAbC-1(C3/V5 targeted, EMD-23223) and pAbC-2(N241/N289 glycan hole targeted, EMD-23224); From left to right (second row) Rh.33104 (Grp1): pAbC-1(N241/N289 glycan hole targeted, EMD-23227) and pAbC-2(N241/N289 glycan hole targeted, EMD-23228); From left to right (third row) Rh.33311 (Grp2): pAbC-1(FP targeted, EMD-23236), pAbC-2(N611-glycan targeted, EMD-23237), and pAbC-5(C3/V5 targeted, EMD-23240). Antibody heavy chain in medium purple and light chain in pink, Env gp120 in dark gray and gp41 in light steel blue throughout Figure 3. Each panel includes a schematic representation (created with BioRender.com).

The development of stabilized Env ectodomain constructs also synergized with development of improved methods for broadly neutralizing antibody (bNAb) isolation through high throughput single-cell B-cell receptor (BCR) amplification and novel soluble Env selection tools together with the culture of memory B cells that allowed the identification and isolation of more potent and broader bNAbs (Walker et al., 2009; Walker et al., 2011; Huang et al., 2012) Single particle cryo-EM analysis uses recombinant soluble stabilized trimeric Env ectodomain constructs that mimic the native spike structure. The clade A BG505 SOSIP.664 has been used as the gold standard HIV native-like engineered Env trimer immunogen allowing the high-resolution structure determination of the Env trimer (Sanders et al., 2013b) in complex with various naturally-elicited antibodies (Figure 3A) and vaccine-elicited antibodies (Figure 3B) to reveal how antibodies can access Env sites of vulnerability by overcoming the barriers posed by the glycan shield, variable loops, and conformational masking (Ananthaswamy et al., 2019; Saunders et al., 2019; Seabright et al., 2020; Yang et al., 2022; Banach et al., 2023; Henderson et al., 2023; Holt et al., 2023; Wang et al., 2023; Saunders et al., 2024; Wiehe et al., 2024; Williams et al., 2024). Another clade B virus improved version of Env with MPER region lacking the cytoplasmic tail (Env ΔCT) was reported that contributed insights into a more native-like Env structure (Lee et al., 2016).

Cryo-EM has impacted the ongoing HIV-1 vaccine development on multiple fronts, that include the facilitation of the design of native-like functional Env immunogens to elicit broadly neutralizing antibodies (bNAbs), high resolution definition of antibody epitopes to establish conserved sites of vulnerability, visualization of antibody virus co-evolution and evaluation of vaccine-induced antibodies for their precision of targeting the desired sites (Lyumkis et al., 2013; Saunders et al., 2019; LaBranche et al., 2019; Torrents de la Peña et al., 2019; Antanasijevic et al., 2020; Cottrell et al., 2021; Roark et al., 2021; Henderson et al., 2023; Saunders et al., 2024; Wiehe et al., 2024). Structural determination by cryo-EM facilitated the discovery of a novel category of Fab-dimerized glycan reactive antibodies that target a Env glycan cluster (Williams et al., 2021). A rapid EM-based polyclonal epitope mapping (EMPEM) method has facilitated direct high resolution structural determination of antibody epitopes from vaccine sera (Figure 3C) (Bianchi et al., 2018; Antanasijevic et al., 2021).

### 3.2 Advantages of cryo-ET over SPA to study HIV-1 viral infection and assembly

Cryo-electron tomography (cryo-ET) and subtomogram averaging (STA) are techniques to study dynamic biological processes and provide unprecedented structural details at moderately high resolution while conserving spatial arrangement in native environments. Cryo-ET has also broadened its applicability by combining with other methods such as correlative light and electron microscopy (CLEM), cryo-focused ion beam (FIB) milling, ion-abrasion scanning electron microscopy (SEM), or focused ion beam-scanning electron microscopy (FIB-SEM) which reveal a wealth of information with new insights (Felts et al., 2010; Jun et al., 2011; Earl and Subramaniam 2013; Blanco-Rodriguez et al., 2020; Zila et al., 2021). Cryo-ET is a rapidly growing field, with method developments being simultaneously carried out to achieve quicker, better, and more detailed outcomes, namely, denoising (Narasimha et al., 2008) and segmentation to reveal intermediate virus assembly stages (Bartesaghi et al., 2005; Narasimha et al., 2008; Woodward et al., 2015; Ladinsky et al., 2020). A newly developed scalable, end-to-end framework for single particle cryo-ET data analysis enables resolution of conformational heterogeneity *in situ* during on-the-fly preprocessing of tilt series without subtomogram averaging followed by high-resolution refinement and classification (Liu et al., 2023). Despite numerous solved structures of HIV-1 and receptor complexes, several aspects of the HIV-1 entry mechanism remain unresolved and continued advances in structural determination will enable discoveries to further elucidate the HIV-1 entry and fusion mechanism.

## 4 Structural studies on the SARS-CoV-2 S protein

### 4.1 Cryo-EM structure of SARS-CoV-2 spikes of viral variants

The outbreak of severe acute respiratory syndrome coronavirus (SARS-CoV) in 2002 and Middle East respiratory syndrome coronavirus (MERS-CoV) in 2012 had already shown that coronaviruses can cross the species barrier to emerge as highly pathogenic viruses (Lu et al., 2015). More recently, SARS-CoV-2 caused a global pandemic that started in December 2019 and was subsequently named COVID-19 (Zhu et al., 2020). The highly infectious disease is characterized by fever, acute respiratory illness, and pneumonia. The SARS-CoV-2 surface spike (S) glycoprotein has a trimeric structure similar to SARS-CoV and many other enveloped pathogenic viruses. This spike consists of three S1-S2 heterodimers that bind the cellular receptor angiotensin-converting enzyme 2 (ACE2) and mediate fusion of the viral and cellular membranes through extensive conformational change (Song et al., 2018; Lan et al., 2020; Wrapp et al., 2020). The pre-fusion S protein consists of S1 subunit that includes N-terminal domain (NTD), receptor-binding domain (RBD) and S2 subunit that includes fusion peptide (FP), heptad repeat 1 (HR1), heptad repeat 2 (HR2), transmembrane (TM) and cytoplasmic tail (CT) (Wang et al., 2020; Wrapp et al., 2020).

The first SARS-CoV-2 trimeric spike high-resolution structures were determined soon after the pandemic begun (Walls et al., 2020; Wrapp et al., 2020). Previous research on the MERS-CoV and SARS-CoV S proteins facilitated by cryo-EM were instrumental in the discovery of the pre-fusion S protein stabilizing two proline (2P) mutations used in several vaccines that were rapidly developed to combat the COVID-19 pandemic (Kirchdoerfer et al., 2016; Pallesen et al., 2017; Walls et al., 2020). Single particle cryo-EM analysis was pivotal to characterizing the structures and conformations of the SARS-CoV-2 S protein and in the structure-based design of engineered S protein constructs (Cai et al., 2020; Henderson et al., 2020; Hsieh et al., 2020; Lu et al., 2022; Shi et al., 2023). A 6P version of pre-fusion SARS-CoV-2 S protein that exhibited better immunogenicity than the S-2P is a component of next-generation stabilized spike vaccines (Carreño et al., 2023; Zhu et al., 2024). While studies of the pre-fusion S protein dominated the cryo-EM bandwidth, innovative studies on the postfusion conformation of the S protein enabled a comprehensive understanding of the fusion mechanism and the impact of mutations on the process (Yang S. et al., 2022; Shi et al., 2023). Lipid nanodiscs have been shown to play a crucial role in visualizing the membrane interacting regions in postfusion SARS-CoV-2 spike proteins using cryo-EM. The full-length postfusion spike in the lipid bilayer allows for the visualization of structural details in the membrane-interacting regions FP and TM during the final stage of membrane fusion. Additionally, mutations in the fusion peptide proximal region (FPPR) completely inhibit its interaction with FP, subsequently blocking membrane fusion activity (Shi et al., 2023). Another study focused on the SARS-CoV-2 membrane (M) protein, an essential component of the viral assembly when placed on a lipid nanodisc revealed structural details and ion channel activity of the M protein (Dolan et al., 2022).

**FIGURE 4:**
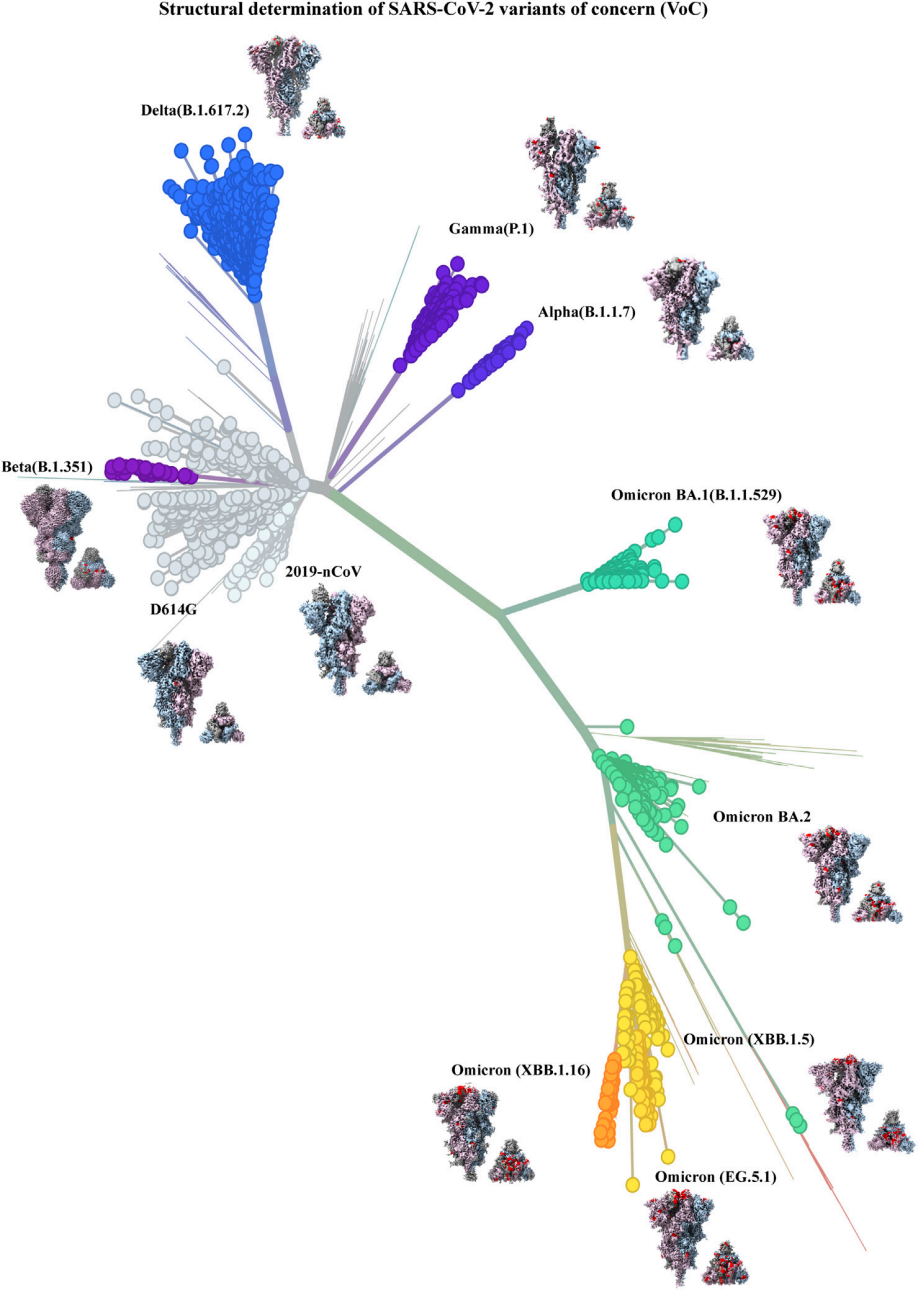
Structural determination of SARS-CoV-2 variants of concern (VoC) (built with nextstrain/ncov). 2019–2024 evolution of SARS-CoV-2 spikes of key emerging variants of concern (VoC): 2019-nCoV (EMD-21375), D614G (EMD-22825), Alpha (B.1.1.7) (EMD-14229), Beta (B.1.351) (EMD-27505), Gamma (P.1) (EMD-24987), Delta (B.1.617.2) (EMD-24981), Omicron BA.1 (B.1.1.529) (EMD-25896), Omicron BA.2 (EMD-26433), Omicron (XBB.1.5) (EMD-43320), Omicron (XBB.1.16) (EMD-42860), Omicron (EG.5.1) (EMD-44000). All cryo-EM structures (side and top view) of spike show the three monomers in dark gray, light steel blue and thistle colors and mutations in spike are shown in red color.

Early in the COVID-19 pandemic, a D614G substitution was identified in the SD2 subdomain of the SARS-CoV-2 S protein that was retained in all subsequent variants (Korber et al., 2020). Cryo-EM analysis of D614G substitution revealed an altered spike conformation with higher proportion of RBD up states, that was accompanied by enhanced sensitivity to neutralization (Yurkovetskiy et al., 2020; Zhang et al., 2021b; Gobeil et al., 2021; Weissman et al., 2021). Subsequently several variants of SARS-CoV2 emerged and swept local and global populations, with some major lineages designated as variants of concern (VOCs) due to increased transmissibility, disease severity, resistance to neutralizing antibodies elicited by vaccines, or reduced efficacy of treatments (Carabelli et al., 2023; Aleem, Akbar Samad and Vaqar, 2024). Figure 4 shows the evolution of SARS-CoV-2 through the course of the COVID-19 pandemic and highlights the role played by cryo-EM in our rapid understanding of evolving SARS-CoV-2 variants (Wrapp et al., 2020; Zhang et al., 2021a; Gobeil et al., 2021; Cerutti et al., 2022; Mannar et al., 2022; Stalls et al., 2022; Wrobel et al., 2022; Mannar et al., 2024; Zhang et al., 2024). The Omicron variant is currently circulating worldwide and has evolved into several sublineages that have successively swept global populations (Parsons and Acharya, 2023). Cryo-EM studies were instrumental in defining the altered structure of the Omicron spikes relative to previous variants and in elucidating how these changes impacted SARS-CoV-2 biology (Cerutti et al., 2022; Gobeil et al., 2022; Mannar et al., 2022; Stalls et al., 2022).

**FIGURE 5:**
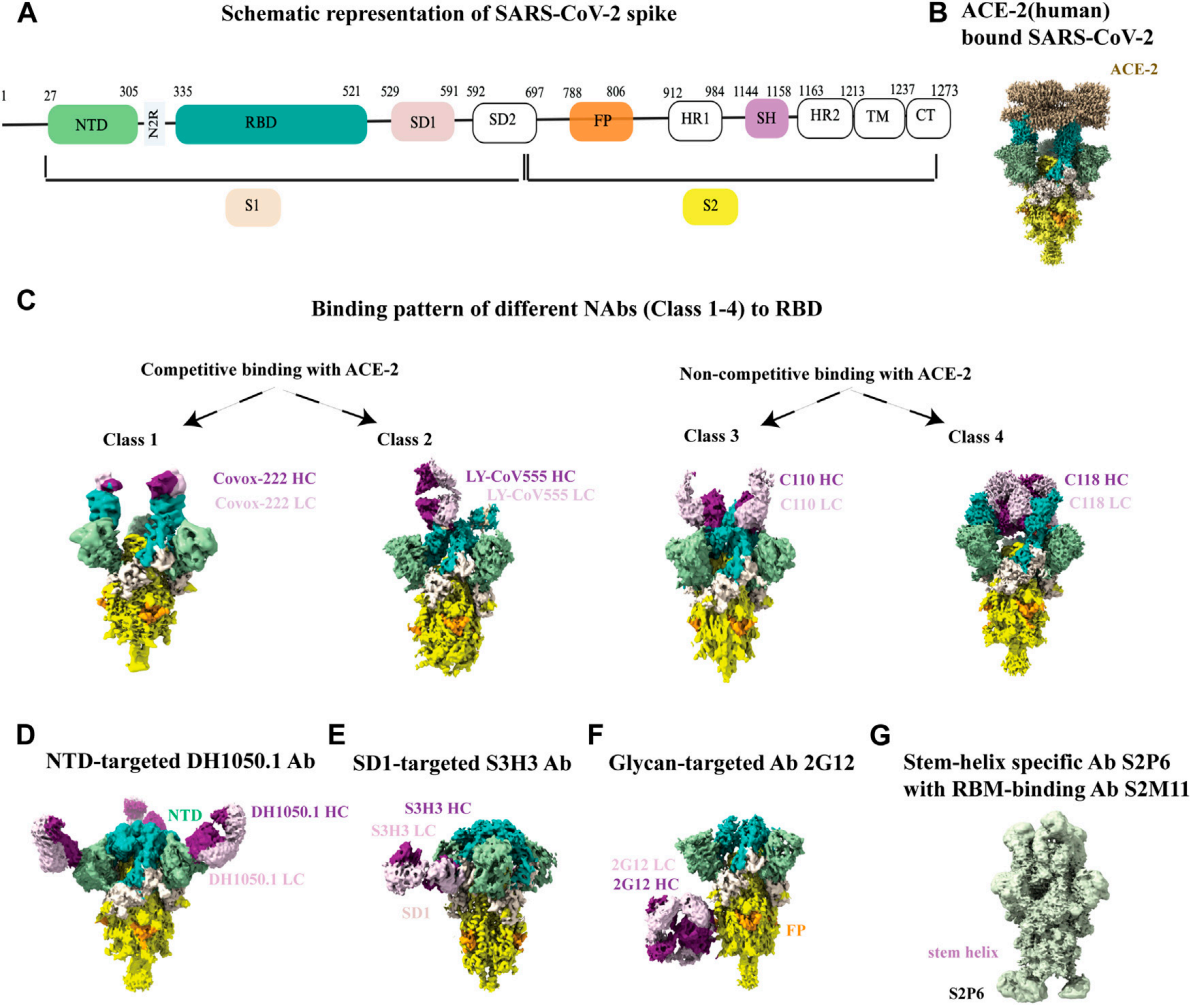
Cryo-EM studies of SARS-CoV-2 spikes and its interaction with antibodies. **(A)** Schematic representation of SARS-CoV2 spike with its different parts color labeled with S1 (antique white), S2 (yellow), N-terminal domain (NTD, dark sea green), receptor binding domain (RBD, sea green), SD1 (misty rose), fusion peptide (FP, orange) and stem helix region (pink violet).The same color scheme was maintained throughout Figure 5 except 5G. Antibody heavy chain is shown in purple and light chain is in thistle color. **(B)** ACE-2 bound (human) SARS-CoV-2 XBB.1.5 spike (EMD-43324). **(C)** Binding patterns of different NAbs (class 1, EMD-13869), (class 2, EMD-23156), (class 3, EMD-22732), (class 4, EMD-24504) to RBD. **(D)** Binding of NTD-targeted Ab DH1050.1 (EMD-23277). **(E)** Binding of SD1-targeted Ab S3H3 (EMD-32564). **(F)** Binding of glycan-targeted Ab 2G12 (EMD-23094). **(G)** Binding of stem-helix specific Ab S2P6 along with RBM-bound S2M11 (EMD-24533).

Overall structure of the SARS-CoV-2 spike protein is schematically illustrated in Figure 5A. Recognition and binding to the host cell receptor is the initial step of virus entry into the host cell. Figure 5B depicts the cryo-EM structure of the SARS-CoV-2 XBB.1.5 spike protein in complex with human ACE2 (Mannar et al., 2024). Cryo-EM structural studies revealed that ACE2 can only interact with the RBD (receptor-binding domain) of the soluble S glycoprotein trimer when the soluble trimer is in RBD-up conformation. The structures also show that that the N-terminal domain (NTD) slightly shifts outward after RBD-ACE2 binding, but no substantial changes occur in the S2 subunit (Xiao et al., 2021b; Jackson et al., 2022). Furthermore, the cryo-EM structure of the soluble S trimer in complex with ACE2 at serological and endosomal pH levels of 7.4 and 5.5, respectively, showed no difference in stoichiometries. Visualization of the soluble S trimer without a ligand at different low pH levels, ranging from pH 5.5 to pH 4.0, revealed a reduction in conformational heterogeneity, ultimately resulting in all RBDs adopting a down conformation (Zhou et al., 2020). Most studies with the ACE2 receptor have used the ACE2 ectodomain, although a cryo-EM structure of the full-length ACE2 protein bound to the receptor-binding domain (RBD) of the SARS-CoV-2 S-glycoprotein was reported (Yan et al., 2020).

For SARS-CoV-2, although the dominant cellular receptor is ACE2, there exists an ACE2-independent pathway for cell entry. Accessory proteins on the membrane or alternative receptors, such as CD147 (extracellular matrix metalloproteinase), NRP1 (Neuropilin-1, a transmembrane protein), CD26 (also known as DPP4, dipeptidyl peptidase IV), AGTR2 (Angiotensin II receptor type 2), C-type lectins, LDLRAD3 and few others, have been implicated in mediating virus attachment and entry in the absence of ACE2 (Alipoor and Mirsaeidi, 2022; Lim et al., 2022). The spike protein is the target of almost all neutralizing antibodies found in the sera of a recovering person or elicited by vaccines. Throughout the pandemic, cryo-EM was also instrumental in visualizing antibody binding to the SARS-CoV-2 spike, enabling our understanding of the immune response in atomic level detail and informing the development of vaccines and therapeutics (Barnes et al., 2020; Jette et al., 2021; Jones et al., 2021; Li et al., 2021; Pinto et al., 2021; Williams et al., 2021; Hong et al., 2022; Liu et al., 2022) RBD-directed neutralizing antibodies are grouped in four different classes (1-4) based on the cryo-EM structural analysis of ACE-2 competitive or non-competitive binding site (Figure 5C). There are other neutralizing antibodies available that binds to the N-terminal domain (NTD) (Figure 5D), highly conserved epitopes in SD1 region (Figure 5E), high mannose glycan cluster of S2 subunit (Figure 5F), and stem-helix region in S2 domain (Figure 5G). Non-competing antibody cocktails could be a potential solution to prevent mutational escape from antibody regimens. While escape mutants arose in the presence of a single antibody, no resistance mutants were detected in the presence of non-competing or partially competing RBD binding site antibodies, (Baum et al., 2020). Aside from antibody-bound structures, the cryo-EM structure of the SARS-CoV-2 spike protein with linoleic acid (LA) bound to the hydrophobic pocket of the RBD to lock the spike in a stable conformation and occlude its most immunogenic epitopes, highlights the ability of cryo-EM to determine structures of small molecules bound to spike at high resolution and yield important mechanistic insights (Toelzer et al., 2023).

### 4.2 Cryo-ET of SARS-CoV-2 virus reveals intact virion, the mechanisms of receptor binding and antibody neutralization

**FIGURE 6:**
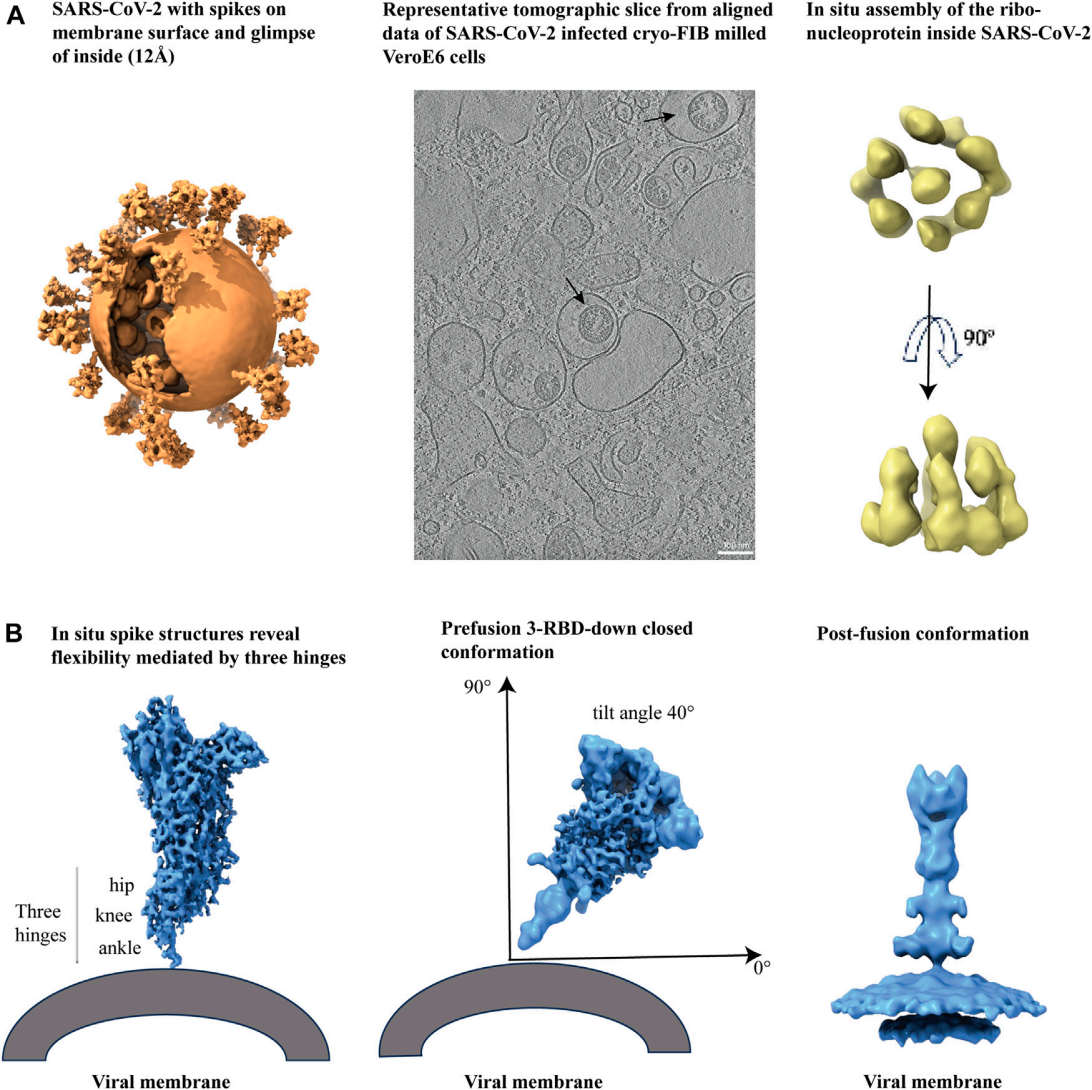
Cryo-electron tomography of membrane bound SARS-CoV-2 spikes. **(A)** From left to right, SARS-CoV-2 virus with spikes from subtomogram averaging (12Å) put back on membrane surface and glimpse of inside, EMD-30430, a representative tomogram slice of SARS-CoV-2 infected and cryo-FIB milled VeroE6 cells (black arrows indicate virions), EMD-11865, and *in situ* assembly of the ribonucleoprotein inside SARS-CoV-2 virus, EMD-30429. **(B)** From left to right, *In situ* spike structures reveal flexibility mediated by three hinges, EMD-11223, prefusion spike viewed at a 40° angle from the normal axis on membrane in a 3 RBD-down conformation, EMD-30426, and post-fusion conformation, EMD-30428.

Single-particle cryo-EM analysis has accelerated the process of determining structures of soluble SARS-CoV-2 S protein constructs. However, to visualize the spike structure and distribution on mature viruses in their natural environment, cryo-ET and subtomogram averaging (STA) have been used for *in situ* analysis (Figure 6A, left panel) (Yao et al., 2020). Cryo-ET studies of infected Vero E6 cells have revealed structural insights into the viral replication compartment, including the budding and assembly mechanism of virions, and the knowledge of packaging of extra-large genomes inside virion (Figure 6A, middle panel) (Klein et al., 2020). Furthermore, cryo-ET studies of SARS-CoV-2 provide information on the spatial arrangements of RNA filaments. Additionally, subtomogram averaging of vRNP complexes has shown a preferred stacking orientation of RNPs (Figure 6A, right panel) (Klein et al., 2020; Yao et al., 2020).

The S protein on the surface of intact SARS-CoV-2 shows significant conformational diversity, including at its receptor binding site and in its spatial arrangement relative to the viral membrane. This provides a fundamental understanding of how the S protein interacts with the ACE2 receptor and neutralizing antibodies (Ke et al., 2020). The spike protein has shown some flexibility due to the presence of three hinges (Figure 6B, left panel) that are shielded by glycans and few such spikes together can interact with the host cell (Turoňová et al., 2020). Hinge glycans are mostly conserved and are held responsible for the bending of membrane-anchored spikes (Figure 6B, middle panel) (Yao et al., 2020). Additional insights on glycan shielding of the SARS-CoV-2 spike have been provided by molecular dynamics (MD) simulation studies (Casalino et al., 2020). A study on human coronavirus NL63 (HCoV-NL63) spikes reported greater oligomannose glycan shielding compared to SARS-CoV-2 spikes indicating better evasion from the host immune response for the former. Furthermore, molecular MD simulation of the HCoV-NL63 full length spikes show the function of hinge glycans in regulating spike flexibility (Chmielewski et al., 2023). The prefusion spike has intrinsic instability and therefore convert to postfusion state (Figure 6B, right panel) with spontaneous S1 dissociation (Cai et al., 2020; Yao et al., 2020).

The pre-fusion and post-fusion states of SARS-CoV-2 spike structures have been successfully resolved using cryo-EM SPA and cryo-ET techniques. However, the transient intermediate states of the fusion process remain to be detected. Cryo-ET is a powerful technique that can help capture these transient states. For example, a study reported the results of capturing extended and partially folded intermediate states of S2 by using an antiviral lipopeptide entry inhibitor (Marcink et al., 2022). Additionally, time-resolved cryo-EM and other complementary structural techniques hold the promise of enabling visualization of transient intermediates that occur along the viral entry pathways at rapid timescales (Harder et al., 2023; Bennett et al., 2024).

## 5 Bacteriophages, the bacterial viruses against infectious diseases including multidrug-resistant (MDR) strain-mediated diseases

**FIGURE 7:**
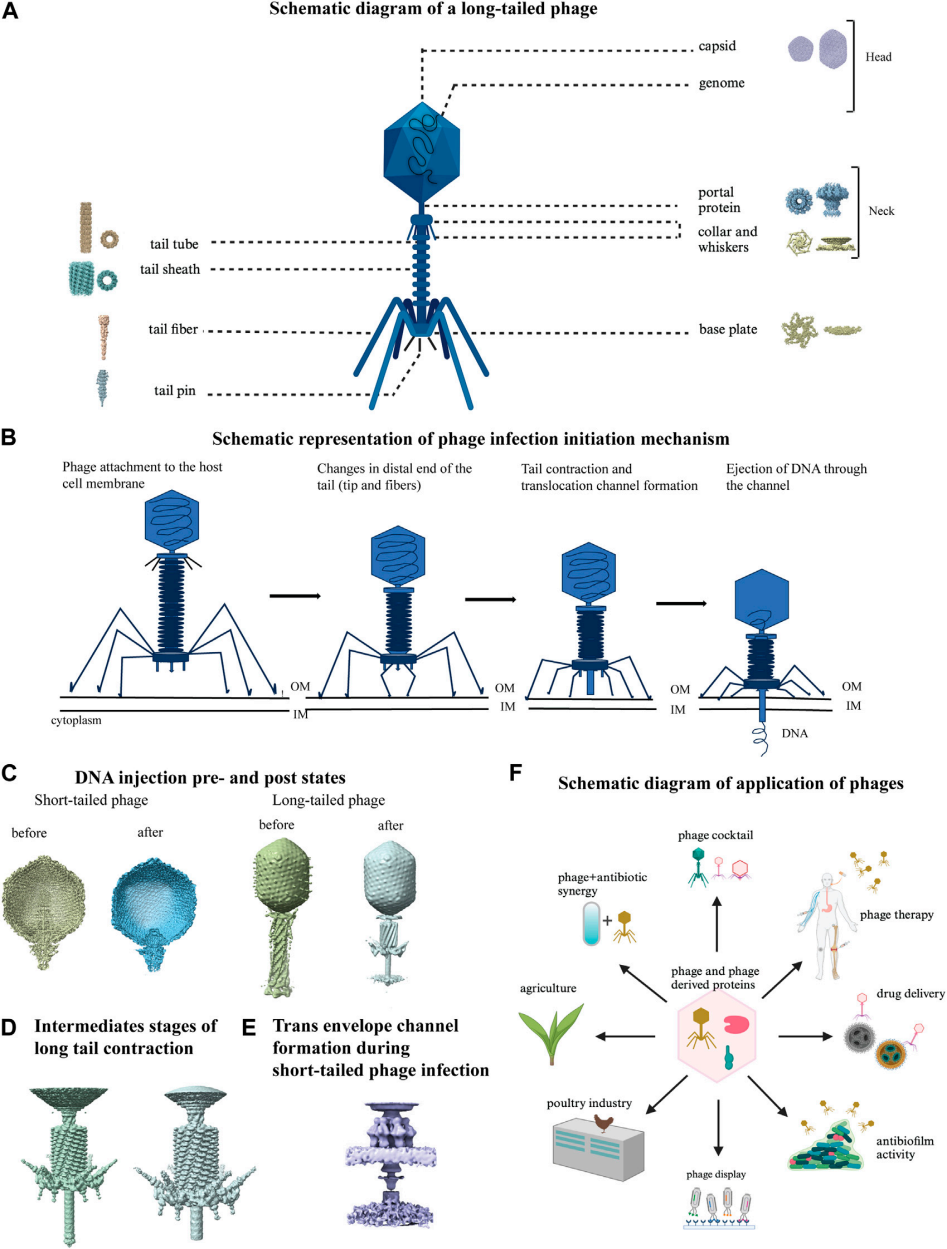
Structural studies on bacteriophages using cryo-EM. **(A)** Schematic diagram of a long-tailed bacteriophage with its parts labeled. Structure of different parts of bacteriophages were solved either individually or partially together and a representative cryo-EM map is shown for each part: capsid (EMD-40228), portal protein (EMD-43145), collar and whiskers (EMD-5528), tail tube (EMD-29354), tail sheath (EMD-36127), base plate (EMD-8064), tail fiber (EMD-34968), tail pin (EMD-35824) in figure A (created with BioRender.com). **(B)** Phage infection initiation mechanism is shown with a schematic model. **(C)** DNA pre injection and post injection states are shown in short tailed (left, EMD-31315, 31318) and long-tailed phages (right, EMD-2774, 6082). **(D)** Close view of detailed structural changes during intermediate stages of long tail contraction (EMD-1089, 1086). **(E)** Cryo-EM map of trans envelope channel formation during short-tailed phage infection (EMD-9006). **(F)** Schematic diagram of various application of phages (created with BioRender.com).

Bacteriophages infect bacteria and are an emerging therapeutic tool to combat MDR bacteria (Viertel et al., 2014; Taati et al., 2020; Hibstu et al., 2022). Bacteriophages are the most abundant entity in any ecosystem where their host bacteria are present (Clokie et al., 2011; Romero-Calle et al., 2019). There are two types of bacteriophages: lytic or virulent, and lysogenic or temperate. Lytic phages, which have a short replication cycle and large burst size, are often preferred for therapeutic purposes (Fernández et al., 2019). Cocktails of phages can also be effective in treating bacterial infections and minimizing phage resistance (Chan et al., 2013; Abedon et l. 2021; Duc et al., 2023). Better and broader application of phages requires a deeper understanding of their structural components in different conformations and how the components function (Helen and Elena, 2019). In 1959 the first image of negatively stained T2 phage was produced (Brenner et al., 1959). EM has contributed to the detection of bacteriophage, their morphology and classification, and structure determination. Advances in cryo-EM technology have allowed researchers to study phage structures by single particle analysis and cryo-electron tomography in unprecedented detail (Helen and Elena, 2019; Belnap et al., 2021). Phages usually have a capsid head, head-tail interface (neck) and tail part, and the latter part participates in the recognition of host receptors, determining host range, and initiating infection (Chaban et al., 2015). A schematic representation of a long-tailed phage is shown in Figure 7A with different parts labelled with representative cryo-EM structures (Fokine et al., 2013; Yap et al., 2016; Ayala et al., 2023; Yang et al., 2023; Zhu et al., 2023; Mukherjee et al., 2024; Sonani et al., 2024; Wang et al., 2024). The maturation process of bacteriophage capsid head from immature procapsid takes place through a series of transient intermediates that have been extensively studied by cryo-EM (Ionel et al., 2011; Veesler et al., 2012; Huet et al., 2019; Wang et al., 2022). The capsid assembly and maturation process is closely connected to genome packaging inside phage capsid and cryo-EM has made substantial contributions in understanding this process (Rao and Black, 2010; Bayfield et al., 2019; Orlov et al., 2022; Hawkins et al., 2023). The major unit of head-tail interface is a dodecameric portal protein located in the vertex connected to the tail. It is a well-studied component of tailed dsDNA bacteriophages showing their diversity by cryo-EM with continuous improvement in the resolution (Orlova et al., 1999; Cerritelli et al., 2003; Dedeo et al., 2019; Javed et al., 2021). Phage tail recognizes the host cell through its receptor-binding protein and sends a signal to the phage head that are followed by a cascade of conformational change throughout phage tail and the formation of a channel to transfer the genome from phage capsid to the host cytoplasm (Figure 7B) (Cuervo et al., 2013; Arnaud et al., 2017; Wang et al., 2024). The visualization of infection and genome delivery mechanism of phages started in early 2000 using cryo-ET (Böhm et al., 2001; Liu et al., 2011). This is still a poorly understood process that involves sequential conformational changes in phage structural components mainly the tail components which are now a heavily studied area enabled by current advances of cryo-ET (Fu et al., 2011; Guerrero-Ferreira et al., 2019). With the high-resolution snapshots of the entire event of phage-host interactions, it was possible to capture pre and post infection/injection structures of short and long-tailed phages (Figure 7C) (Hu et al., 2015; Chen et al., 2021), high-resolution well studied structures of intermediate stages of long-tail contraction (Figure 7D) (Leiman et al., 2004), formation of transmembrane channel during short-tailed phage infection (Figure 7E) (Wang et al., 2019). All these have helped to propose a general mechanism of phage infection to destroy host bacteria (Hu et al., 2015; Nováček et al., 2016; Wang et al., 2019).

Phage therapy, which is over a century old, has been revived to combat the current crisis of antimicrobial resistance (AMR). Thanks to advanced technologies, such as high-throughput sequencing, synthetic biology, and genetic engineering, phages are now being used for other applications apart from antibacterial activities (Chen et al., 2019; Olsen et al., 2020; Lenneman et al., 2021). Once the high-resolution structural details and sequencing data for a new phage are available, genetical modification or phage structural engineering can be done to create a better version for therapeutic or industrial purposes (Gibb et al., 2021; Guo et al., 2021). By understanding the structure of phages, they can be applied in potential capsid-based delivery systems (e.g., drug, antigen), bio-nanotechnology, and can combat infectious diseases more effectively. For example, a study demonstrated that the icosahedral bacteriophage capsid can be used as a system to carry host cell hemagglutinin in an arrangement that blocks the entire influenza viral surface by binding to it in a defined multivalent mode, which inhibits the infection process (Lauster et al., 2020). Additionally, a phage display-based model was used to study the diversity within the receptor-binding domain (RBD) of SARS-CoV-2 Env proteins (Pérez-Massón et al., 2024). There are many other applications of phages beyond phage therapy or drug delivery. In Figure 7F, the multitude of applications of phage is described in a schematic presentation.

## 6 Discussion

Cryo-EM has had a significant impact on the field of structural virology and helped understand infection process of bacterial viruses to animal/human viruses. With the structural knowledge in hand, virus entry and infection mechanism models were proposed. The detailed understanding of the mechanism of HIV-1 virus attachment and entry into host cells using advanced cryo-EM technology has revealed previously unknown target sites for therapeutic intervention. There still remains a constant need for more effective and safe drugs and new targets to overcome the growing resistance of a highly mutating virus HIV-1 (Bean, 2005; Adamson and Freed, 2009). Identification of host-related targets can be a potential solution as the virus may find it difficult to develop resistance against the drug (Puhl et al., 2019). There are small molecule compounds identified through computer-aided molecular docking tools or high throughput screening to block the virus-CD4 receptor interaction (early phase), coreceptor entrance (advanced phase), and membrane fusion (final phase) (Briz et al., 2006; Yu et al., 2014; Xiao et al., 2020). APOBEC3G (A3G) is a key component of the human innate immune system to defend against invading viruses, whereas the viral infectivity factor (Vif) of HIV-1 virus helps them to neutralize cellular defenses. A recent report provides valuable information on APOBEC3G-Vif interaction, a prime event of viral immune suppression to overcome host defenses (Li et al., 2023). As a result, this has become a potential target for new therapeutics against HIV-1. Single Particle cryo-EM analysis was used to study the interaction of the HIV-1 capsid with the first-in-class capsid-targeting antiretroviral compound GS-6207 (lenacapavir) (Highland et al., 2023).

The COVID-19 pandemic caused by SARS-CoV-2 and its variants caught much scientific attention. While whole genome analysis and structural studies were the forefront at identifying and characterizing new variants, antibody isolation techniques coupled with cryo-EM structural determination accelerated our knowledge of how the immune system responds to SARS-CoV-2 and led to the development of antibody-based therapeutics. While therapeutic antibodies rapidly became ineffective against new variants, new computational tools coupled with structural analysis were employed to successfully redesign antibodies to bind the evolved variants (Desautels et al., 2024).

Antibiotic-resistant bacterial infection is a growing public threat that needs immediate attention. Phage therapy is a powerful alternative strategy to address the increasing number of drug-resistant bacterial infection and biofilm-mediated infection where lytic phages are deployed to infect and destroy the drug-resistant bacterial cells. Bacteriophages are plentiful in all ecosystems and play a role in controlling bacterial populations and evolution. The use of phages to address antibiotic resistance is gaining attention within the “One Health” framework, which encompasses not only humans, but also other animals (Kittler et al., 2017; Garvey, 2020). The most promising side of phage therapy is that there are no harmful effects so far reported. Still phage therapy is uninviting for many reasons such as immune response, selection of effective phages, lack of knowledge to setup well-developed clinical trials, and occurrence of phage resistance (Fernández et al., 2019; Hitchcock et al., 2023; Noor et al., 2024). In this review we mostly focused our discussion on structural studies of phages by cryo-EM and how this technique contributed to further understanding phage structures and proposing model for infection, assembly and egress (Guerrero-Ferreira and Wright, 2013; Linares et al., 2020; Conners et al., 2023). Current maps and models derived from cryo-EM data also showed the interior of the phage structure to reveal the highly ordered hexagonal packaging arrangement of DNA (Bayfield et al., 2019; Orlov et al., 2022).

## 7 Future epidemics and other emerging infectious disease threats: potential of cryo-EM

The COVID-19 pandemic has shown the aftermath of a pandemic causing severe damage to the health system, death toll, and socioeconomic condition of any country. At the same time, it showed us that extensive international collaboration in health science sectors can have a substantial impact on controlling the healthcare crisis and other collateral damages caused by a pandemic. Along with the advances in cryo-EM, other innovative approaches are being developed and integrated with cryo-EM for a comprehensive understanding of various events of physiological processes. The integrative approaches include CLEM (correlative light electron microscopy), FIB-SEM(focused ion beam scanning electron microscopy), Mass spectrometry, and time-resolved TEM (Tuijtel et al., 2019; Amann et al., 2023; de Beer et al., 2023; Lorenz 2024). These integrated cryo-EM approaches will contribute to overcoming the challenges of infectious disease studies especially where viral protein purification is difficult or for visualizing rapid time-scale events. With the current knowledge of vaccine technology and programs such as Defense Advances Research Program Agency (DARPA), Coalition for Epidemic Preparedness Innovations (CEPI) and Advanced Research Projects Agency for Health (ARPA-H), precautionary measures and infrastructures built to handle future pandemics and any new emerging disease threats are needed for pandemic preparedness (Sempowski et al., 2020). Modern state-of-the-art cryo-EM modalities combined with advanced image processing and other modern technologies, including AI-based structure prediction tools, have the potential to accelerate and enable structure-based design of interventions to combat infectious diseases (Jumper et al., 2021; Schoehn et al., 2023).
